# *Brucella suis* biovar 1 infection in a dog with orchitis in Germany

**DOI:** 10.3389/fvets.2023.1233118

**Published:** 2023-08-03

**Authors:** Sophie Aurich, Juliane Schneider, Hanka Brangsch, Ad Koets, Falk Melzer, Christa Ewers, Ellen Prenger-Berninghoff

**Affiliations:** ^1^Department of Veterinary Science, Institute for Hygiene and Infectious Diseases of Animals, Justus Liebig University, Giessen, Germany; ^2^Tierärztliche Klinik für Kleintiere am Kaiserberg, Duisburg, Germany; ^3^Institute of Bacterial Infections and Zoonoses, Friedrich-Loeffler-Institute, Federal Research Institute for Animal Health, Jena, Germany; ^4^Department of Bacteriology, Host-Pathogen Interaction and Diagnostics, Wageningen Bioveterinary Research, Lelystad, Netherlands

**Keywords:** canine brucellosis, zoonosis, *Brucella suis* biovar 1, epididymitis, reproductive disease, whole genome sequencing, genotyping, raw meat diet

## Abstract

In 2021, a case of canine brucellosis diagnosed in a dog with orchitis was presented to a veterinary practice in Germany. Serological testing excluded *Brucella* (*B*.) *canis* as a causative agent, but molecular analysis revealed the presence of *B. suis* biovar 1. Since biovar 1 is not endemic in Europe and the dog had no history of travel to endemic areas, a comprehensive epidemiological investigation was conducted using whole genome sequence data to determine the source of infection. We describe the clinical progress of the animal and the potential infection of a veterinary clinic employee. The findings highlight the importance of considering less common *Brucella* species as possible causes of canine brucellosis. The data also emphasize that it is quite challenging to identify *Brucella* species in a routine diagnostic laboratory and to conduct epidemiological investigations to unveil possible transmission routes.

## 1. Introduction

Brucellosis primarily manifests in the reproductive system of various terrestrial and aquatic mammals. It has been associated with abortion and other reproductive as well as chronic disorders, thus inflicting significant economic losses and posing a threat to human health due to its zoonotic potential (1). It is caused by the genus *Brucella*, a coccoid, Gram-negative, aerobic to microaerophilic rod that is slow-growing and requires complex nutrients for culturing (2). Of the six classical *Brucella* species, namely, *B. melitensis* (sheep and goats), *B. abortus* (cattle), *B. suis* (swine, hares, and reindeer), *B. canis* (dogs), *B. ovis* (sheep), and *B. neotomae* (rodents), only the first four are well-known relevant terrestrial zoonotic pathogens (3–5). For humans, *B. melitensis* is considered the most pathogenic species, followed by *B. suis, B. abortus*, and *B. canis* (5–10).

Canine brucellosis is most commonly caused by *B. canis* and is mainly noticed by infertility and reproductive failure in dog breeding kennels (11, 12). It has a high affinity for the testes, epididymides, prostate, and uterus but also colonizes the lymphatic organs, eyes, and spinal column. Typical clinical signs in females are infertility in form of conception failure, embryo resorption, late-time abortion, and vaginal discharge after abortion or parturition; in males, epididymitis, orchitis, or prostatitis may be noticed. Uveitis, discospondylitis, and meningitis may occur as symptoms independent of the reproductive tract or as long-term effects (12–15). The main path of infection is sexual transmission (11, 16). A study concerning the incidence of *B. canis* in Europe between 2011 and 2016 detected 5.4% of serologically positive animals. However, only samples from dogs with suspected *B. canis* infections were tested. Therefore, the actual incidence of canine brucellosis due to *B. canis* in Europe remains unknown (17).

In addition to *B. canis*, the causal agents of canine brucellosis have also been sporadically identified as *B. suis, B. abortus*, and *B. melitensis* (18). Concerning the five biovars of *B. suis*, biovars (bv) 1–3 are the predominant cause of porcine brucellosis and manifest in the reproductive tissues, causing reproductive disorders and infertility (19). Bv 1 exhibits the highest zoonotic potential and virulence concerning humans and pigs (8, 20): In regions where *B. suis* bv 1 is endemic, such as Australia or the United States, infection of humans and dogs associated with hunting or feeding raw meat of feral pigs/wild hogs has been documented (21–27). While *B. suis* bv 1 is most prevalent in Southern Asia, North and South America, and Oceania, *B. suis* bv 2 is present in European brown hare populations in Europe and frequently found in wild boars and domestic pigs (28, 29). Unlike bv 1, bv 2 rarely infects humans (30).^1^

In Europe, there are few reports of *B*. *suis* bv 1 infections in humans (20, 31–34); in dogs, infection with *B. suis* bv 1 was reported in Berlin, Germany, in 1978 and in the Netherlands in 2016, both presumably caused by consumption of uncooked meat (32, 33).

The objective of this report is to provide a comprehensive account of the clinical presentation, microbiological characteristics, and molecular analysis of a case of canine brucellosis caused by *B. suis* bv 1 in Germany, including investigations of the source by molecular typing.

## 2. Methods

### 2.1. Case history

In July 2021, a 2-year-old male intact Rhodesian Ridgeback was presented to a primary care veterinary practice in North Rhine-Westphalia for apathy and inappetence. The dog showed lameness, a reluctance to lie down, and bilateral painful swollen testes. Suspecting an infective orchitis, the dog was treated with amoxicillin/clavulanic acid (12.5 mg/kg bid). After 2 days of antibiotic therapy, the dog was neutered because clinical signs did not improve. At surgery, purulent exudation from the testes and a thickened spermatic cord were noted. Antibiotic therapy was modified by administering amoxicillin/clavulanic acid (20 mg/kg bid) and marbofloxacin (2 mg/kg qd).

Four days after the dog was neutered, it was referred to a veterinary clinic due to fever, vomiting, and weight loss. Abdominal sonography revealed ascites and an enlarged, inhomogeneous prostate. Blood analysis resulted in leukocytosis (28/μl), neutrophilia (21.56/μl), monocytosis (4.38/μl), hypoalbuminemia (2.2 g/dl), hypochloremia (105 mg/dl), and a high level of C-reactive protein (0.85 mg/dl). Analysis of urine showed high levels of leukocytes, erythrocytes, protein (30 mg/dl), high specific gravity (1050), and presumably the presence of coccoid bacteria. The ascitic fluid was revealed to be a septic exudate with a specific gravity of 1,026, total protein of 30 g/dl, cell count of 107,200 cells/μl, the presence of phagocytosed intracellular coccoid bacteria, activated mesothelial cells, and degenerated neutrophils. During the following diagnostic laparotomy the next day, 2 L of a brownish exudate was collected, peritonitis was noted throughout the abdominal cavity, and multiple abscesses were found in the left spermatic cord. Subsequently, the left spermatic cord was resected, and abdominal lavage was performed. Following surgical intervention, a tentative diagnosis of brucellosis was established, and the dog was placed in the isolation ward. Antibiotic treatment was adjusted to trimethoprim/sulfamethoxazole (5 mg/kg qd) and doxycycline (10 mg/kg qd). Clinical symptoms improved, and the dog had a full recovery within 7 days.

### 2.2. Sampling

During neutering at the primary care veterinary practice, purulent exudate from the testes and spermatic cord was collected for microbiological examination. This sample was sent to the Institute of Hygiene and Infectious Diseases of Animals at the Justus Liebig University in Giessen, Germany (IHIT). In addition, during a posterior laparotomy performed at the veterinary clinic, ascitic fluid and abscess material were collected. These samples were sent to an external diagnostic laboratory for microbiological analysis. Blood samples taken during laparotomy were sent to the IHIT for serological testing for *B. canis*.

To determine the source of infection, the local veterinary department obtained samples of the dogs' feed provided by the owners, a commercially available raw meat diet consisting of beef, horse, salmon (origin unknown), and kangaroo sourced from Australian farms. Only salmon and kangaroo meat were available at the time of the investigation and were subsequently subjected to testing for *Brucella* species at the corresponding Chemical and Veterinary Analytical Institute.

### 2.3. Microbiological investigation

For initial microbiological analysis, the sample was streaked on standard nutrient agar (Oxoid, Wesel, Germany) containing 5% defibrinated sheep blood (blood agar) and on water-blue metachrome-yellow lactose agar, according to Gassner (Sifin Diagnostics GmbH, Berlin, Germany). The plates were incubated at 37°C for 48 h in ambient air. For bacterial enrichment, the sample was cultivated for 24 h at 37°C in standard I nutrient broth (E. Merck KG, Darmstadt, Germany) and streaked on 5% sheep blood agar and Gassner agar. For the detection of microaerophilic bacteria, the sample was additionally streaked on brain-heart infusion agar (Oxoid) and incubated for 5 days in 10% CO_2_ at 37°C. Schaedler agar (Becton Dickinson GmbH, Heidelberg, Germany) and Zeissler agar (E. Merck KG) were incubated for 72 h at 37°C under anaerobic conditions in a jar using the AnaeroGen™ gas sachets (Oxoid). After 48 h, abundant growth (>200) of small, non-hemolytic, shiny colonies was evident on blood agar. Similar colonies were also observed on brain-heart-infusion agar. Identification with matrix-assisted laser desorption time-of-flight mass spectrometry (MALDI-TOF-MS, Bruker Daltonics, Bremen, Germany) using the standard MBT Compass reference library (version 10.0.0.0) and the Security-Relevant (SR) Library was performed.

For further identification, the isolate was sent to the reference laboratory of the World Organization for Animal Health (WOAH) and National Reference Laboratory (NRL) for *brucellosis in animals*, the Institute of Bacterial Infections and Zoonosis at the Friedrich-Loeffler-Institut, Jena, Germany. Pathogen identification and biovar typing were conducted by the so-called Bruce-Ladder and New Bruce-Ladder polymerase chain reaction (PCR) (35, 36).

The dogs' feed was analyzed using cultural detection methods and real-time multiplex PCR according to the published protocols for the detection of brucellosis in cattle, pigs, sheep, and goats of the NRL (37).

### 2.4. Serological investigation

Serological testing for *B. canis* was performed with *B. canis*-specific antigens (in-house antigen) using the tube agglutination test (TAT) at the IHIT. In addition, serological tests [slide agglutination test (SAT), complement fixation test, and Rose Bengal Test] with *B. abortus* antigens (IDEXX Montpellier SAS, Montpellier, France) cross-reacting for *B. abortus, B. suis*, and *B. melitensis* were performed at the NRL for *brucellosis in animals*.

### 2.5. Genomic characterization

#### 2.5.1. DNA isolation and whole genome sequencing

DNA was extracted from pure cultures using the High Pure PCR Template Preparation Kit (Roche Molecular Systems, Pleasanton, CA, United States). For short-read sequencing by Illumina technology, a genomic library was prepared using the NexteraXT kit (Illumina Inc., San Diego, CA, United States), which was sequenced on a MiSeq (Illumina Inc., San Diego, CA, United States) in paired-end mode. For obtaining a closed genome, the DNA was additionally sequenced by Nanopore technology on a MinION Mk1B device (Oxford Nanopore Technologies Ltd., Oxford, England). The corresponding genomic library was prepared with the Ligation Sequencing kit (SQK-LSK109) and barcoded using the EXP-NBD 104 kit (Oxford Nanopore Technologies Ltd., Oxford, England). This library was run on a R9.4.1 flow cell for 24 h.

#### 2.5.2. *De novo* assembly and annotation

Reads from both Illumina and Nanopore technologies were used for combined *de novo* genome assembly using microPIPE (38) with basecalling in super-accuracy mode. The assembly quality statistics were assessed using QUAST version 5.0.2 (39), and the annotation was carried out using Bakta version 1.6.1 with database version 4.0 (40).

#### 2.5.3. Genome comparison and genotyping

For determining the origin of the isolate, NCBI's Sequence Read Archive (SRA) and RefSeq genome databases were browsed (accessed in January 2023) for *B. suis* bv 1 sequences (Supplementary Table 1). Furthermore, MLVAbank [https://microbesgenotyping.i2bc.paris-saclay.fr/; accessed February 9, 2023 (41)] was searched for multiple locus variable number of tandem repeats analysis (MLVA) profiles with similarity to the investigated isolate displaying a maximum of three alleles difference.

The average nucleotide identity of the newly assembled genome compared to other *B. suis* bv 1 strains deposited in the NCBI RefSeq database was assessed using fastANI version 1.1 (42). To exclude that the isolate was identical to the *B. suis* bv 1 vaccine strain S2, an *in silico* PCR using a script by Egon A. Ozer (version 0.5.1) (https://github.com/egonozer/in_silico_pcr) was conducted with primers IclRP1 (5′-TGGCAAGAGCGGTTTCAG-3′) and IclRP2 (5′-TCCAAGGTCGGCTACGAA-3′) (43). *In silico* MLVA was carried out using the MISTReSS (https://github.com/Papos92/MISTReSS) as described by Sacchini et al. (44). Based on the differences in alleles, a minimum spanning tree was calculated using GrapeTree version 1.0 (45) with the implemented MSTreeV2 algorithm. In addition, core genome multilocus sequence typing (cgMLST) using Ridom Seqsphere+ version 7.7 (46) and the scheme by Abdel-Glil et al. (47) was conducted. Foreign strains for which exclusively raw reads have been deposited on NCBI were assembled using Shovill version 1.0.4 (https://github.com/tseemann/shovill) with the SPAdes assembler and the option “- - trim”. CgMLST allelic distances were used for the calculation of a minimum spanning tree as implemented in Ridom Seqsphere+. Typing based on core genome single nucleotide polymorphisms (cgSNPs) was performed by using Snippy version 4.6.0 (https://github.com/tseemann/snippy) using *B. suis* bv 1 strain 1330 (GCF_000223195.1) as reference. In this analysis, SRA data was included. The cgSNP alignment was used for the calculation of a phylogenetic maximum likelihood tree using RAxML version 8.2.12 (48) with the GTRGAMMA model. The tree was visualized using FigTree version 1.4.3 (http://tree.bio.ed.ac.uk/soft-ware/figtree/).

## 3. Results

### 3.1. Microbiological analysis

Initial identification of the isolate, denominated 21RB23181, with MALDI-TOF-MS revealed *Brucella melitensis*, yielding a score of 2.39. Although species identification must be considered questionable due to the fact that the commercial database only provides reference spectra for *B. melitensis*, it has to be assumed that genus identification is correct. Therefore, after differentiation as *Brucella* sp., the results were immediately forwarded to the local veterinary department, and the sample was handled in compliance with official protective measures (49, 50). The isolate was tested by the NRL for *brucellosis in animals* by PCR, which identified 21RB23181 as a *B. suis* bv 1 strain.

The sample of ascitic fluid and abscess material sent to an external diagnostic laboratory did not result in the detection of *Brucella* sp. Similarly, the remaining samples of salmon and kangaroo meat obtained by the local veterinary department tested negative for *Brucella* sp.

### 3.2. Serological analysis

Serological testing of a blood sample for *B. canis* using the TAT assay yielded a negative result (<40 IU/ml). However, when tested with antigens specific to the *B. melitensis, B. abortus*, and *B. suis* group, the sample showed a positive result (SAT 841 IU/ml, complement fixation test 1,189 SensE/ml, and positive in the Rose Bengal test).

### 3.3. Genomic analysis

#### 3.3.1. Genome characterization and similarity

Using a combined assembly approach of Illumina and Nanopore reads, the genome of 21RB23181 could be assembled to completion at a mean coverage of 271×. The genome consisted of two circular contigs of 2,107,952 and 1,207,151 bp with an average GC content of 57.25% and 3,113 predicted coding sequences.

The highest ANI values were observed for *B. suis* bv 1 strains Human/AR/US/1981 (99.9958% ANI) and VBI22 (99.9949% ANI), both isolated in the United States, and vaccine strain S2 (99.9946% ANI) isolated in China. *In silico* PCR yielded a negative result for the S2-specific primer pair, thus ruling out the possibility that 21RB23181 was the vaccine strain.

#### 3.3.2. Genotyping using allele-based methods

**Figure 1:**
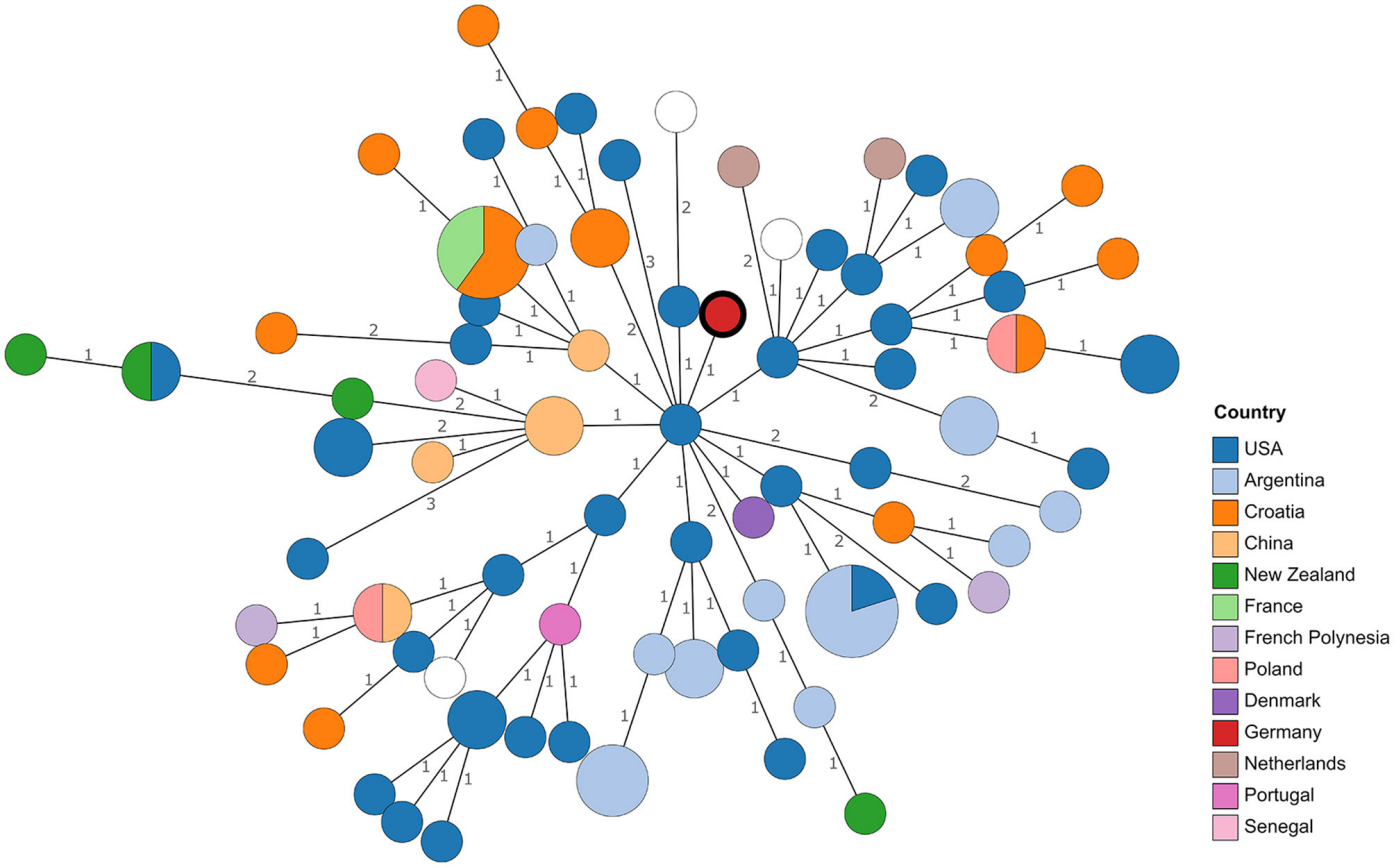
Minimum spanning tree based on MLVA profile differences. Numbers on branches indicate allele differences. Leaves are colored according to the origin of the strains. The leaf representing isolate 21RB23181 has a bold margin. For empty leaves, the origin is unknown.

Although WGS offers the possibility to compare strains at the nucleotide level, the lack of genome sequences from reported isolates necessitates resorting to comparing MLVA profiles, despite the lower resolution. Profiles published in MLVAbank and in literature (see Supplementary Table 2) were included. Remarkably, in the resulting minimum spanning tree (Figure 1), no clustering of strains according to their origin could be observed. Especially strains from Croatia, the United States, and Argentina were scattered in the tree. With the exception of one allele, the MLVA profile of 21RB23181 matched that of a strain isolated in Denmark in 1987 (BCCN#87-85) and strain B93-0078, which originated from cattle in the United States in 1993. Despite the closer geographic location, the distance to *Brucella* isolates from a dog (WBVR_2016) and a hare in the Netherlands (WBVR_2017) displayed higher differences with four differing alleles each.

**Figure 2:**
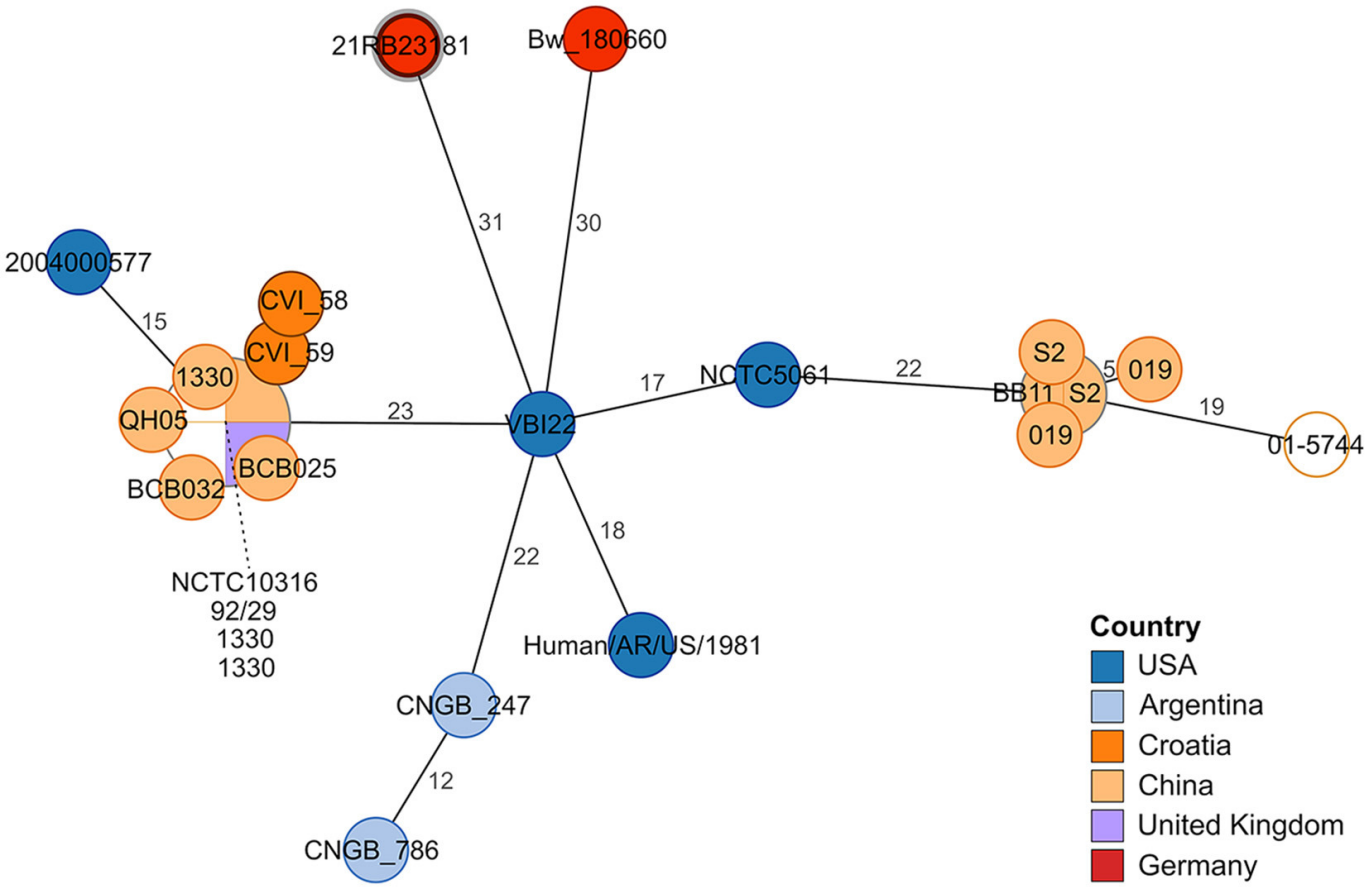
Minimum spanning tree based on allelic distances determined by cgMLST analysis. The numbers on the branches indicate allele distances. The leaves are colored according to the strain's origin. The leaf representing isolate 21RB23181 has a bold margin. For better readability, the names of clustering leaves are connected to the leaves by a dashed line. For empty leaves, the origin is unknown.

In the cgMLST analysis, 21RB23181 exhibited at least 31 allele differences compared to other *B. suis* bv 1 strains (Figure 2). The strain VBI22 isolated from cattle in the United States displayed the highest degree of similarity. In this analysis, a *B. suis* bv 1 isolate from a human brucellosis case in Germany in 2018 (Bw_180660) was also included. However, it displayed 45 allele differences from the German dog isolate, so no connection between these two cases could be inferred.

#### 3.3.3. Genotyping using SNPs

**Figure 3:**
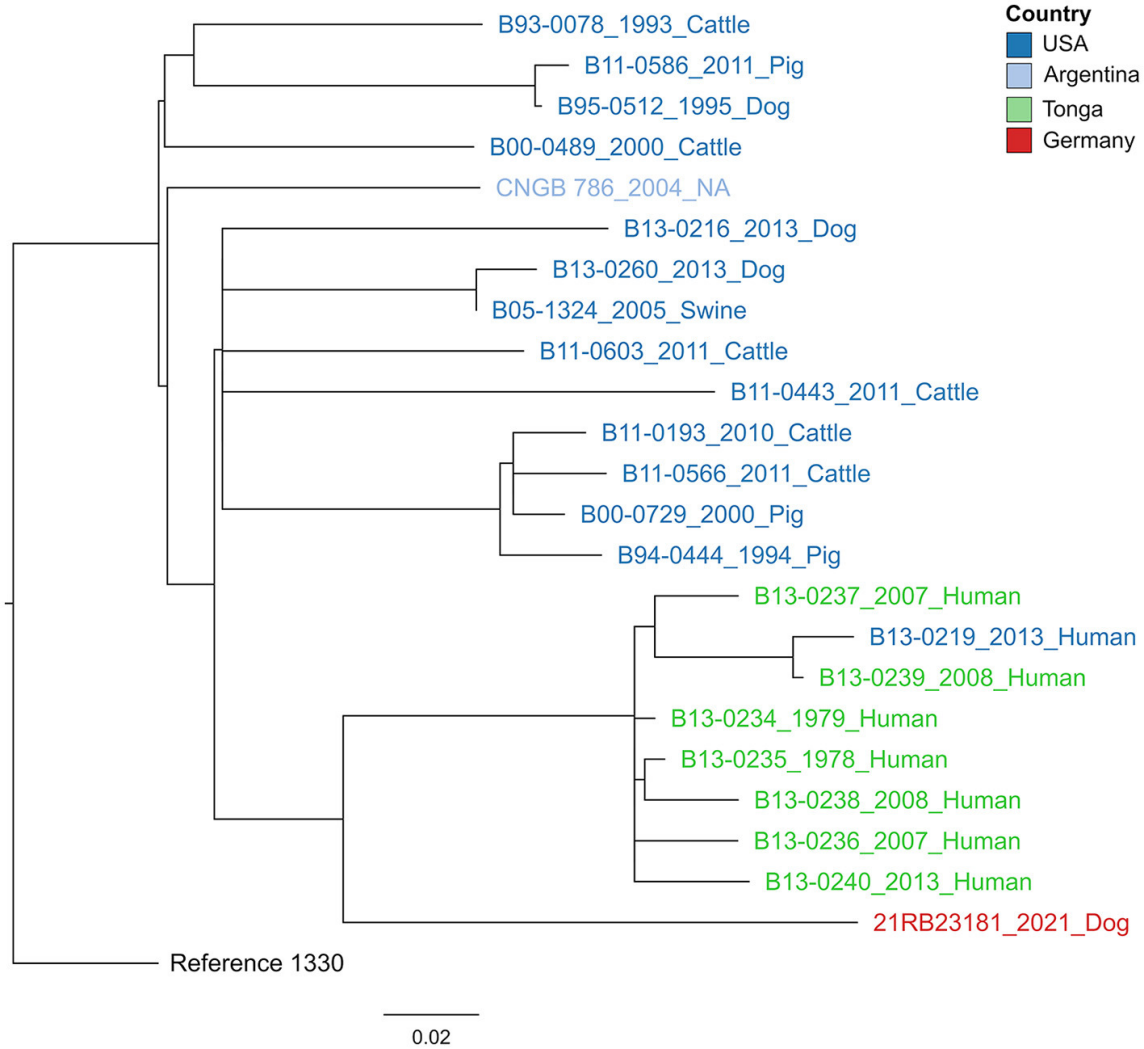
Maximum likelihood tree based on core genome SNP alignment. The tree is rooted in the reference strain *B. suis* bv 1 strain 1330. The colors of the labels give the origin of the isolates. The year of isolation and host are given after the strain names. The bar indicates the number of base substitutions per site.

In a cgSNP approach (Figure 3), 21RB23181 was compared to *B. suis* bv 1 isolates for which raw read data were available (Supplementary Table 1). In total, 476 core genome SNPs were called. The isolate 21RB23181 was most similar to strains originating from Tonga, with the highest SNP identity to a strain isolated in 1979 from a human in Tonga (69 SNPs). However, differences between strains from the United States and Argentina were only slightly higher, ranging between 77 and 96 SNPs. Based on this result, the genomes of the strains from Tonga were assembled using the raw sequencing data, and cgMLST analysis was repeated (Supplementary Figure 1). The allelic profiles of two strains from Tonga exhibited higher concordance with 21RB23181 than the US American strain VBI22, differing in 27 alleles, but still, this difference is not much smaller than that of the US American strain. These strains from Tonga are partially also represented in the MLVA tree; however, in the MLVAbank database, their origin is given as New Zealand (B13-0234, B13-0236, B13-0237, and B13-0239). In the MLVA, 21RB23181 differs in three alleles from the strains originating from Tonga.

### 3.4. Feasible measures concerning possible transmission

Immediately after the detection of *Brucella* sp., the zoonotic risk for the involved veterinary practices and the microbiological laboratory was assessed. The employees with increased risk due to close contact with the animal or handling the microbiological sample and the pure culture before identification of the isolate were informed and subjected to serological control. One staff member of the primary care veterinary practice who had been in close contact with the animal showed a slightly increased IgM antibody value of 21 U/ml (reference range, <15 U/ml) using *Brucella* IgM and IgG antibody enzyme-linked immunosorbent assay (ELISA) (Virion/Serion, Würzburg, Germany) ~14 days after the first contact with the dog. IgG antibody and immune-capture-agglutination test values (bestbion dx GmbH, Cologne, Germany) remained within the normal range. The affected employee also reported symptoms like fatigue and night sweats lasting for about a week. Symptoms improved rapidly while receiving doxycycline (100 mg bid for 3 months). The follow-up serology 6 weeks later still showed slightly elevated IgM values of 19 U/ml but no increase in IgG antibody values. The *Brucella* immune-capture-agglutination test resulted negative again. However, no attempts were made to isolate the bacterium by direct culture. To date, 1.5 years later, the employee continues to be symptom-free.

## 4. Discussion

This report illustrates the first notified *B. suis* infection in a dog in Germany since 1978. The infected individual was an intact male Rhodesian Ridgeback suffering from orchitis with symptoms including fever, testicular swelling and pain, abscessed spermatic cord, free abdominal fluid due to peritonitis, anorexia, and weakness. Microbiological analysis of an abscessed testis after neutering revealed the presence of *Brucella* sp., identified by PCR as *B. suis* bv 1.

In areas where *B. suis* bv 1 is endemic in wildlife, canine infection with the pathogen is not uncommon. The incidence of infection in dogs has been reported to increase in certain regions of the United States and Australia (51–53). In most of these cases, exposure to feral pigs through hunting or the consumption of raw feral pig meat was associated with the infection. In Europe, only two cases of *B. suis* infection in dogs have been reported to date: In 1978, *B. suis* bv 1 was detected in two male dogs in Berlin, Germany, showing fever and orchitis/epididymitis. Since they were kept only in urban surroundings and bv 1 is not endemic in Germany, the dog feed (raw meat from Eastern European countries) was suspected as the source of infection (33). The second case reported was a dog infected with *B. suis* bv 1 in the Netherlands in 2016. It had no history of hunting or travel to *B. suis* bv 1 endemic countries but was fed uncooked hare meat imported from Argentina. Via PCR, *B. suis* bv 1 was detected in these hare carcasses and characterized *in silico* by MLVA and MLST. Both samples of the dog and the meat showed high similarity in MLVA (32).

Unfortunately, in the case presented here, it was not possible to determine the source of infection. The patient was neither a hunting nor a breeding dog, although contact with infected vaginal fluid or urine from bitches could not be excluded. The dog, raised in Germany, had a history of travel to the Netherlands but had never been to *B. suis* bv 1 endemic regions. Since cases of *B. suis* bv 1 infection were associated with consumption of raw meat, this route of transmission is probable but uncertain, especially since *Brucella* sp. could not be detected in the analyzed meat. This result is limited by the inability to test all types of meat and the uncertainty regarding whether the tested meat was from the same batch consumed during the time of infection. Given the variable incubation period of *Brucella* infection, which can range from 2 weeks to several months, some time may have elapsed from infection to the onset of symptoms (11).

Transmission of *B. suis* from dogs to humans has not yet been clearly demonstrated. A single report from the United States suggests a *B. suis* infection in a woman as a result of handling aborted canine fetuses without gloves (54). However, in the case reported here, the serological results of the staff member do not ultimately prove infection. An increase in IgM antibodies alone may indicate a non-specific reaction and not necessarily an infection. The symptoms presented by the affected employee were characteristic of a *Brucella* infection but were not specific and therefore inconclusive. Unfortunately, no cultural or direct evidence by PCR has been obtained, leaving the possibility of human infection as speculative but noteworthy, especially since a case of *B. suis* bv 1 infection in a human without any serological response has also been reported (55). In Europe, only two human cases of infection with *B. suis* bv 1 were described to the authors' knowledge: A Spanish medical waste treatment plant worker was infected with *B. suis* bv 1 in 2014, probably after an accidental puncture with a contaminated needle (34). Presumably as a consequence of private meat processing, a German became infected with *B. suis* bv 1 in 2018 (20).

Standard serological tests for suspected canine brucellosis will only detect *B. canis*, as there is no serological cross-reaction between *B. canis* and *B. suis* since they have different antigenic characteristics. *B. canis* and *B. ovis* carry a rough lipopolysaccharide (LPS) without the most external antigen, the O-polysaccharide, while all other *Brucella* species are smooth *Brucella* strains with the O-polysaccharide present in the LPS (2, 56). This can lead to false-negative results for infection with *Brucella* species other than *B. canis*, so direct culture is preferred. Still, accurate identification of isolates using the standard method of MALDI-TOF MS is challenging, as only reference spectra for *B. melitensis* are available in the database. Given that this organism is highly clonal and demands methods with high discriminatory potential, whole genome sequencing is widely employed for the identification and differentiation of *Brucella* sp. (47, 57). Three years before the presented case, brucellosis was also diagnosed in a dog in the Netherlands, and *B. suis* bv 1 was isolated (32). However, based on the MLVA results, no epidemiological connection could be drawn between both cases. Likewise, the isolate from a human brucellosis case in Germany in 2018 (20) markedly differed from 21RB23181. Determining the geographic origin of the infection source in the presented case is hampered by the lack of comprehensive sequencing data. Regarding Europe, only a limited number of *B. suis* bv 1 WGS data is available, of which 21RB23181 did not show a notable similarity. With regard to the known distribution of *B. suis* bv 1 strains, it can be expected that the strain is not of endemic origin but imported, maybe from the Pacific region (e.g., Polynesia), as SNP typing revealed a higher similarity to strains from Tonga. The fact that MLVA would put 21RB23181 closer to US American isolates as the allelic distance to Tonga strains was comparably high can be disregarded. It was shown for *B. melitensis* that MLVA results can lead to false conclusions regarding strain origin and that WGS-based methods are more appropriate (57).

Despite its recovery, the dog was euthanized after being diagnosed with *B. suis* bv 1, given the high risk of zoonotic transmission. *Brucella* disseminates through direct contact, ingestion, or aerosolization of body fluids. It may be intermittently shed for up to 60 weeks and remains persistent for at least 2 years after inoculation (12). Due to its intracellular nature and periodic bacteremia, antibiotic treatment is often unsuccessful. The most promising therapy involves a combination of tetracyclines (high-dose doxycycline or minocycline for 1–2 months) and aminoglycosides (streptomycin or gentamicin for the first 2 weeks). However, relapses can occur shortly after the discontinuation of the antibiotic. Given the high risk of zoonotic transmission, prolonged shedding, and poor treatment options, euthanasia of affected dogs is recommended (12).

Herewith, we report the detection of the third case of canine brucellosis caused by *B. suis* bv 1 in Europe. Whole genome sequencing was used to determine the phylogenetic relationship of the isolate to other strains, with the aim of tracing the origin of the infection and identifying possible transmission routes. However, due to the limited availability of relevant sequence data, it was not possible to clearly determine the origin of the isolates, but connections to other European cases could be excluded. Although transmission from dogs to humans has not been clearly demonstrated, *B. suis* bv 1 is a highly virulent lineage that frequently infects humans with mild to severe symptoms, posing a threat to dog owners and veterinary personnel. Laboratories should be aware of the difficulties in culturing and serological testing that can result in underdiagnosis of this disease.

## Data availability statement

The datasets presented in this study can be found in online repositories. The names of the repository/repositories and accession number(s) can be found below: https://www.ebi.ac.uk/ena, PRJEB60627.

## Author contributions

EP-B, CE, and FM supervised the project. EP-B, CE, and SA conducted microbiological analyses. HB performed genome sequencing and wrote sections of the manuscript. AK contributed *B. suis* genome data. HB and FM analyzed sequencing data. JS and SA drafted the first version of the manuscript. All authors contributed to the manuscript revision, read, and approved the submitted version.

## Footnotes

^1^ https://www.efsa.europa.eu/de/efsajournal/pub/1144
