# Supplementary material for: Brucella suis biovar 1 infection in a dog with orchitis in Germany
Source: Front Vet Sci. 2023 Aug 3;10:1233118. doi: 10.3389/fvets.2023.1233118 (PMC10435866; doi:10.3389/fvets.2023.1233118)
Supplement: Supplementary file 3 [file Data_Sheet_1.docx]

Supplementary Material

***Brucella suis* biovar 1 infection in a dog with ascending orchitis in Germany**

Sophie Aurich^1*^ †, Juliane Schneider^2^ †, Hanka Brangsch^3^, Falk Melzer^3^, Christa Ewers^1^, Ellen Prenger-Berninghoff^1^

*** Correspondance:** Sophie Aurich: [Sophie.Aurich@vetmed.uni-giessen.de](mailto:Sophie.Aurich@vetmed.uni-giessen.de)

## Supplementary Figures


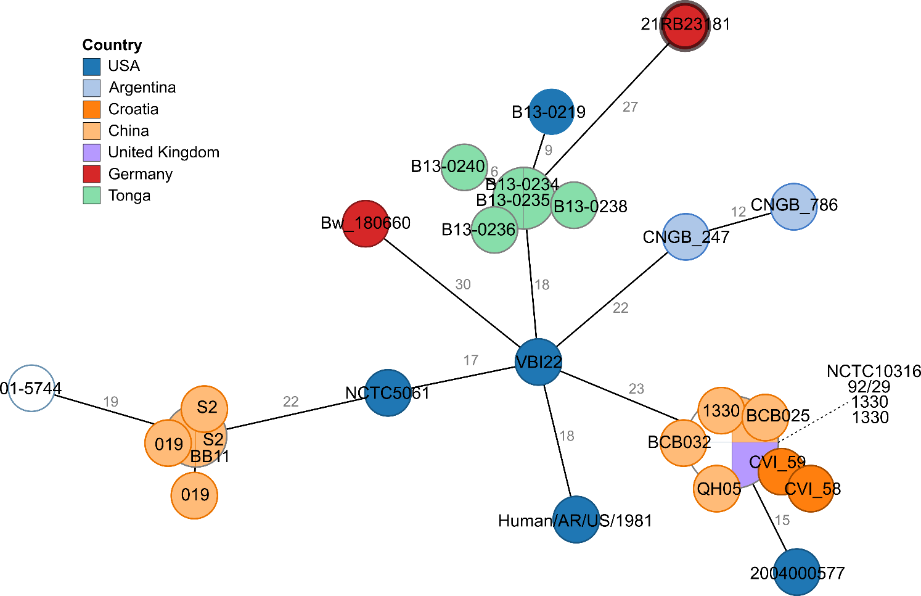


**Supplement Figure 1:** Minimum spanning tree based on allelic distances determined by cgMLST analysis including assemblies of isolates from Tonga. Numbers on the branches indicate allele distances. The leaves are coloured according to the strain origin. The leaf representing isolate 21RB23181 has a bold margin. For better readability, names of clustering leaves are connected to the leaves by a dashed line. For empty leaves, the origin is unknown

## Supplementary Tables

**Supplement Table 1**: Foreign data used in the study for comparison.

**Supplement Table 2**: MLVA profiles used in this study for comparison. Profiles were downloaded from MLVAbank or taken from literature
